# Suggestions for the promotion of evidence-based public health in South Korea

**DOI:** 10.4178/epih.e2017030

**Published:** 2017-07-19

**Authors:** Jong-Myon Bae

**Affiliations:** Department of Preventive Medicine, Jeju National University School of Medicine, Jeju, Korea

**Keywords:** Evidence-based health care, Evidence-based medicine, Decision making, Health policy, Guideline, Communication

## Abstract

Evidence-based public health (EBPH) is defined as public health decision-making based on current best evidence. Debates about the latent tuberculosis infection control program suggested by the Korea Centers for Disease Control and Prevention in 2017 highlight the need to promote EBPH. Three strategies have been proposed, including providing necessary evidence by evaluating policy-evidence gaps; delivering high-quality, relevant, and timely evidence by conducting systematic reviews and adapting public health guidelines; and making value-driven decisions by training and educating public health policymakers.

## INTRODUCTION

In commemoration of Tuberculosis (TB) Prevention Day, on March 24, 2017, the Korea Centers for Disease Control and Prevention (KCDC) announced that 18.78 million people, including those in collective facilities, vulnerable populations, and individuals undergoing health examinations would be screened for latent *Mycobacterium tuberculosis* infections (LTBI) and that prophylactic anti-TB drugs would be provided to those with a positive test result in an attempt to establish a TB-safe country by reducing the incidence of TB to levels comparable to those of developed countries [1]. By May 2017, a claim was raised that there was a lack of scientific evidence for selecting the targets for the LTBI control program [2]. On June 21, the KCDC announced that individuals aged 40 years who were undergoing health examination would be excluded from the LTBI screening program [3].

This is an example of the importance of evidence for scientific validity among the various factors to consider when deciding to conduct public health projects at the national level. Evidence-based public health (EBPH) is based on the use of valid evidence to inform public health-related decision-making [4]. The above case highlights the necessity to promote EBPH for public health program decision-making in South Korea (hereafter Korea). This article examined the definitions of EBPH and evidence generation required for EBPH implementation in order to identify barriers to actively reflecting EBPH in public health decision-making and to suggest methods for the promotion of EBPH in the domestic environment.

## MAIN BODY

### Definition of evidence-based public health

In 1977, Jenicek [4] defined EBPH as ‘the conscientious, explicit, and judicious use of current best evidence in making decisions about the care of communities and populations in the domain of health protection, disease prevention and health maintenance and improvement’. Meanwhile, in 1996, Sackett et al. [5] defined evidence-based medicine (EBM) as ‘the conscientious, explicit, and judicious use of current best evidence from clinical care research in the management of individual patient’. These definitions share the ‘conscientious, explicit, and judicious use of current best evidence in making decisions’, which suggests that the concept of EBPH has been theorized and has evolved in the process of applying the principles of EBM in medicine to the public health domain [6,7]. Both EBM and EBPH emphasize decision-making based on the best valid evidence [8-10]. Thus, the terms ‘evidence-based policy-making’ and ‘evidence-informed decision-making’ have been used instead of EBPH [11,12].

Based on these definitions, EBPH and EBM differ in the following three ways: First, EBM focuses on individual patients, whereas EBPH focuses on community and residents [13]. Second, the EBM intervention is disease treatment, whereas the EBPH intervention is disease prevention and health promotion [14]. Third, EBM implementation is targeted toward medical professionals including physicians, whereas EBPH implementation targets health policymakers responsible for the planning and promotion of public health policies [15].

In 2000, Sackett et al. [16] redefined EBM as ‘the integration of best research evidence with clinical expertise and patient values’. This definition requires that decisions are made by combining the three elements – best evidence, expertise of medical professionals, and patient value – in clinical practice [11]. In this context, in 2004 Kohatsu et al. [17] redefined EBPH as ‘the process of integrating science-based interventions with community preferences to improve the health of populations’. These redefinitions were made with a focus on decision-making, in which two factors, such as best evidence and patient value emphasized in the EBM definition, mutually correspond to science-based interventions and community preference in the EBPH definition [6,7,10,18]. The term ‘scientific intervention’ refers to public health projects in which scientific evidence proves the effectiveness [9]. Value is a factor that determines how to use limited resources [11] and the corresponding community preferences are referred to as ‘community needs’ [17]. For this reason, EBPH decision-making should go beyond evidencebased to be value-based [19], emphasizing the need to communicate with various interest groups with regard to decision-making policies [7].

Unlike the definitions of EBPH that correspond with that of EBM, Brownson et al. [20] defined EBPH as ‘the development, implementation, and evaluation of effective programs and policies in public health through application of principles of scientific reasoning’, focusing on the application of EBPH. In this definition, effectiveness refers to evidence that reflects all benefits, harms, and costs [21].

### Evidence acquisition for evidence-based public health

The common keywords in the three definitions of EBPH by Jenicek [4], Kohatsu et al. [17], and Brownson et al. [20] are ‘scientific evidence’ and ‘decision making’ [8,22]. Especially, reasonable decision-making in public health requires scientific evidence to prove effectiveness [20].

In order to obtain the relevant evidence required for EBPH compared to EBM, the following differences should be considered. First, there is a difference in the study designs that generate evidence. The results of randomized controlled trials which are mainly used in EBM can be rarely utilized in EBPH; thus, the results of ecologic studies, cross-sectional studies, quasi-experimental studies and time-series analyses that have lower scientific power are often used in EBPH [6,13]. Furthermore, expert opinions and case studies are also used as evidence for EBPH [14]. Second, there is a difference in the subjects that utilize the relevant evidence. Medical professionals, especially physicians, independently interpret and apply evidence in EBM, whereas at least four groups – healthcare policy makers, policy practitioners, policy-related interest groups, and public health scholars – are involved in EBPH [6]. Therefore, the evidence required by each subject varies and the degree of acceptance of the evidence also varies [19,23]. Third, there is a difference in the disciplines involved in the development of guidelines to fill evidence-practice gaps. In EBM, the development of clinical practice guideline focused on patient care is based on clinical studies, whereas the development of public health guidelines (PHGs) related to EBPH should involve the results of studies from more diverse disciplines [6,15].

Because of these differences, it is necessary to obtain data from a number of resources and use special strategies to obtain evidence for EBPH implementation [11]. These strategies are summarized as follows: First, it is necessary to operate a dedicated organization for evidence generation. The NICE (https://www.nice.org.uk/) in the UK and the National Evidence-based Healthcare Collaborating Agency (NECA, http://neca.re.kr/) in Korea are representative organizations that pursue EBPH. Second, it is necessary to build a knowledge base to continuously provide information on various risk factors that threaten public health [24]. The Cochrane Library (http://www.cochranelibrary.com/) and Health Research Policy and Systems (https://health-policy-systems.biomedcentral.com/) are representative knowledge base agencies, while the National Center for Medical Information and Knowledge (NCMIK, http://library.nih.go.kr/ncmiklib/) under the KCDC in Korea has the same function. Third, it is necessary to actively use systematic reviews (SR) [11,22] in EBPH to generate evidence to measure the effectiveness and impact of public health programs or to develop PHG [25].

### Factors related to the promotion of evidence-based public health

Because most evidence generated for EBPH is based on the findings of studies conducted in Western countries [6], evidence that applies to domestic public health decision-making is relatively lacking. However, as noted above, the dedicated organization NECA and the NCMIK knowledge base are already available in Korea. Therefore, it is necessary to examine other barriers in addition to the relative lack of evidence as the reasons why EBPH is not utilized actively in domestic situations.

Tricco et al. [25] summarized the barriers to evidence-based decision-making in four dimensions, including attitudes, knowledge, skills, and behavior. Jacobs et al. [26] identified nine barriers to evidence-based decision-making, suggesting that three– lack of skills to develop evidence-based programs, lack of skills to effectively communicate findings to state-level policy makers, and a feeling of the need to be an expert on many issues – were modifiable. Oliver et al. [27] emphasized that the largest barriers to the use of evidence included the lack of timely access to published research findings and the decision-makers’ lack of understanding of the evidence. The first major barrier, lack of timely access to research findings, as indicated in a study by Oliver et al. [27] is similar to the lack of skills to develop evidence-based programs and lack of skills to communicate findings described by Jacobs [26]. The second major barrier, the decision-makers’ lack of understanding of the findings, is concordant with limited expertise. Eventually, the lack of communication between researchers and decision-makers is a barrier to fostering EBPH. Hyder et al. [28] proposed six recommendations for the promotion of EBPH (Table 1), which can be summarized as ways to facilitate communication between researchers and decision-makers [27].

## CONCLUSION AND SUGGESTIONS

In summary, good communication between researchers or evidence generators and administrators or decision-makers should be established first in order to promote EBPH. Regarding the various controversies and subsequent changes in the selection of targets for the 2017 LTBI screening program proposed by the KCDC [1-3], the KCDC, which is responsible for public health-related policy decision-making and implementation, should take the lead in evaluating the level of communication between relevant parties and develop improvement measures for evidence-based decision-making. If the original decision structure was not based on relevant PHG developed based on relevant evidence but was instead based on the opinion of the concerned people such that there was a potential conflict of interest, the situation should be corrected immediately. If there was a lack of evidence necessary for decision-making, it is necessary to conduct pilot projects that target high-risk groups or the underprivileged in order to obtain this evidence before pursuing a public health policy for all citizens [6].

Meanwhile, communication between researchers and administrators is more difficult in EBPH than that between patients and medical professionals in EBM [11] because the EBPH decision-making processes are unique and complex, unlike those of EBM [7]. To overcome this challenge, it is necessary to ensure the stable operation of the affiliated organizations established in accordance with national-level strategies [14]. The Korean government operates the NECA to analyze policy-evidence gaps and generate evidence for public health decision-making [23], the NCMIK to synthesize high-quality evidence such as SR and PHG and deliver evidence timed with decision making [6], and the Korea Health Promotion Institute (www.khealth.or.kr) to provide education and training for decision makers to make value-based decisions [18]. In order to promote EBPH in Korea, it is necessary to further strengthen the leadership network that connects the three organizations [29]. In this regard, we propose to utilize SUPPORT Tools for evidence-informed health Policymaking) operating strategies [12,21] and to actively participate in the Developing and Evaluating Communication Strategies to Support Informed Decisions and Practice Based on Evidence consortium, which already has international cooperation [30].

## Figures and Tables

**Table 1.** Suggested strategies to promote evidence-based public health^1^

Suggested strategies
Strengthening policy-maker demand
Creating formal processes to facilitate dialogue
Improving the packaging of evidence
Enhancing technical capabilities and competencies
Implementing researcher incentives
Recognizing the role of informal relationships

1 Modified from Hyder et al. Health Policy Plan 2011;26:73-82 [28]
