# Supplementary material for: Suggestions for the promotion of evidence-based public health in South Korea
Source: Epidemiol Health. 2017 Jul 19;39:e2017030. doi: 10.4178/epih.e2017030 (PMC5675982; doi:10.4178/epih.e2017030)
Supplement: Supplementary file 1 [file epih-39-e2017030-supplementary.pdf]

Volume: 39, Article ID: e2017030

https://doi.org/10.4178/epih.e2017030

<Perspective>

Suggestions for Promoting Evidence-based Public Health in Korea

배종면

제주대학교 의학전문대학원 예방의학교실

Jong-Myon Bae

Department of Preventive Medicine, Jeju National University School of Medicine, Jeju, Korea

Corresponding authors: Jong-Myon Bae

jmbae@jejunu.ac.kr

Volume: 39, Article ID: e2017030

https://doi.org/10.4178/epih.e2017030

Abstract

Evidence-based public health (EBPH) is defined as public health decision-making based on current best evidence. Debates about the latent tuberculosis infection control program suggested by the Korea Centers for Disease Control and Prevention in 2017 highlight the need to promote EBPH. Three strategies have been proposed, including providing necessary evidence by evaluating policy-evidence gaps; delivering high-quality, relevant, and timely evidences by conducting systematic reviews and adapting public health guidelines; and making value-driven decisions by training and educating public health policymakers.

Keywords: Evidence-based health care, Evidence-based medicine, Decision making, Health policy, Guideline, Communication

Volume: 39, Article ID: e2017030

https://doi.org/10.4178/epih.e2017030

서론

질병관리본부는 2017년 3월 24일 결핵예방의 날의 맞이하여, 결핵 발생률을 선진국 수준으로 감소시키는 결핵안심국가를 만들기 위하여 위해서 집단시설 종사자, 취약계층, 생애주기별 대상자 187만 8천명에게 잠복결핵 감염 (latent *Mycobacterium tuberculosis* infection, LTBI) 여부를 검진하고, 검사 양성자에게는 항결핵제를 예방 투여하기로 발표하였다 [1]. 해당 사업에 적용한 대상자 선정에 의과학적 근거가 부족하다는 주장이 5월 말에 제기되었다 [2]. 그리고 6월 21일 질병관리본부는 40세 건강진단 대상자를 LTBI 검진사업 대상에서 제외한다고 발표하였다 [3].

이상은 국가 차원의 공중보건사업의 수행을 결정할 때 고려해야 할 여러 요소들 중에서 과학적 타당성을 갖춘 근거가 중요함을 보여주는 한 사례이다. 이처럼 공중보건과 관련한 의사결정 과정에 타당한 근거를 기반하는 것을 '근거중심 공중보건 (Evidence-base Public Health, EBPH)라 부르고 있다 [4]. 따라서 상기 사례는 한국의 공중보건 사업 수행의 의사결정에 있어 EBPH의 활성화가 필요함을 제기한 것이다. 본 시론의 목적은 EBPH의 정의 (definition)와 EBPH 수행에 필요한 근거 생성에 관하여 살펴보고, EBPH가 공중보건 의사결정에 적극적으로 반영되지 못하는 요소들을 알아본 다음, 국내 환경에서 EBPH가 활성화되기 위한 제언을 하는 것이다.

Volume: 39, Article ID: e2017030

https://doi.org/10.4178/epih.e2017030

본론

1. 근거중심 공중보건의 정의

1997년 Jenicek [4] 는 EBPH를 '지역사회와 주민에 관련한 건강증진과 질병 예방 영역에 있어 가장 좋은 최신의 근거를 공정하고 명백하고 신중하게 사용하여 의사결정을 하는 것 (conscientious, explicit, and judicious use of current best evidence in making decisions about the care of communities and populations in the domain of health protection, disease prevention, health maintenance and improvement) 이라고 정의하였다. 그런데 1996년 Sackett 등[6]이 EBM을 'The conscientious, explicit, and judicious use of current best evidence from clinical care research in the management of individual patient.' 라 정의한 것과 비교해 볼 때, '가장 좋은 최신의 근거를 공정하고 명백하고 신중하게 사용하여 의사결정을 하는 것'이란 점에서 공통점을 갖는다. 이는 의료계에서 제기된 근거중심의학 (Evidence-based Medicine, EBM)의 원리를 공중보건 영역으로 적용하는 과정에서 EBPH 가 이론화되고 발전되어 왔다는 점을 시사한다 [6, 7]. EBM과 EBPH 모두 최선의 타당한 근거에 따라 의사 결정을 하겠다는 것을 강조하고 있다 [8-10]. 이 점 때문에 EBPH 대신에 근거중심 정책결정 (Evidence-based policy making) [11], 근거정보 의사결정 (Evidence-informed decision

Volume: 39, Article ID: e2017030
https://doi.org/10.4178/epih.e2017030

making)이란 용어 등이 제시되었다 [12].

위의 EBPH와 EBM 정의 간의 차이점들을 살펴 볼 때, 다음 세 가지에서 상호 비교가 된다. 첫째, 적용 대상으로 EBM은 개별 환자인 반면, EBPH는 지역사회와 주민들이다 [13]. 둘째, 실행 개입 (intervention)으로 EBM은 질병치료인 반면, EBPH는 질병예방과 건강증진이다 [14]. 셋째, 시행 주체로 EBM은 의사를 포함한 의료진인 반면, EBPH는 공중보건 정책을 기획하고 추진하는 보건의료 정책당국 (health policy makers)이다 [15].

한편 Sackett 등은 2000년에 EBM을 'The integration of best research evidence with clinical expertise and patient values' 로 재정의 하였다 [16]. 이 정의가 의미하는 바는 임상치료에 있어 최선의 근거 – 의료진의 전문성 – 환자의 가치 의 3가지 요소를 합쳐서 의사결정을 하자는 점이다 [11]. 이에 맞추어 2004년 Kohatsu 등은 EBPH를 '공중보건의 향상을 위하여 과학적 개입과 지역사회의 선호도를 통합하는 과정' ('the process of integrating science-based interventions with community preferences to improve the health of populations.')으로 정의하였다 [17]. 이는 의사결정에 초점을 두고 재 정의한 것으로, EBM에서 강조한 최선의 근거와 환자 가치 두 요소가, EBPH에서는 과학적 개입과 지역사회 선호도로 상호 대응되어 있다 [6, 7, 10, 18]. 여기서 과학적 개입이란 용어에는 효과 (effectiveness)를 입증하는 과학적 근거를 가진 공중보건 사업을 뜻한다 [9]. 가치 (value)란 제한된 자원을

Volume: 39, Article ID: e2017030

https://doi.org/10.4178/epih.e2017030

어떻게 활용할 것인가를 결정하는 요소인데 [11], 이와 대응하는 지역사회 선호도 (preferences)는 주민의 요구도 (community needs)란 의미로도 제시된다 [17]. 이런 이유로 EBPH의 의사결정은 근거중심 (evidence-based)을 넘어 가치기반 (value-based)에서 이루어져야 하며 [19], 결정할 정책과 관련한 여러 이익집단 등과 의사소통이 필요함을 강조한다 [7].

이처럼 EBPH의 정의를 EBM과 대응하여 정의해 온 것과 달리, Brownson 등 [20]은 EBPH의 적용에 초점을 두고 '과학적 추론 원칙을 적용하여 공중보건영역에서 효과적인 사업과 정책을 기획, 수행, 평가하는 것' (the development, implementation, and evaluation of effective programs and policies in public health through application of principles of scientific reasoning) 이라 정의하였다. 이 문구 중 효과 (effectiveness)란 편익 (benefit), 유해 (harm), 비용 (cost)을 모두 반영한 근거를 뜻한다 [21].

2. 근거중심 공중보건을 위한 근거 확보

앞서 언급한 EBPH의 3가지 정의들 - Jenicek [4], Kohatsu [17], Brwonson [20]- 에서 공통적인 키워드는 '과학적 근거'와 '의사결정' 이다 [8, 22]. 특히 합당한 의사결정을 위해선 효과성을 입증하는 과학적 근거를 확보하는 것이 필수적이다 [20].

Volume: 39, Article ID: e2017030
https://doi.org/10.4178/epih.e2017030

EBM과 비교해서 EBPH에 필요한 관련 근거들의 확보를 위해서는 다음 몇 가지 차이점들을 고려해야 한다. 첫째, 근거를 생성하는 연구 설계에서 차이가 있다. EBM에서 주요하게 다루는 무작위배정 임상시험 (randomized controlled trial)의 연구결과들을 EBPH에서는 거의 활용할 수 없어서, 과학적 설득력이 더 낮은 생태학적 연구 (ecologic studies), 단면연구 (cross-sectional studies), 유사실험연구 (quasi-experimental studies), 시계열분석 (time-series analyses) 등의 연구 결과를 활용할 경우가 많다 [6, 13]. 나아가 전문가 의견 (expert opinion), 사례보고 (case study) 등을 근거로 삼기도 한다 [14]. 둘째, 관련 근거를 활용할 주체에서 차이가 있다. EBM에서는 의료진, 특히 주치의란 독자적인 주체가 근거를 해석하고 적용하지만, EBPH에서는 최소한 4 군의 주체들 – 보건의료 정책결정자, 정책수행자, 정책관련 이익집단, 공중보건학자-이 관여한다 [6]. 따라서 주체들마다 요구하는 근거들이 다양하고, 생성된 근거의 수용 정도도 각각 다르다 [19, 23]. 셋째, 근거와 시행 간의 간극 (Evidence-Practice gaps)을 메꾸기 위한 지침 (guideline) 개발에 있어 관여하는 학문 영역에서 차이가 있다. EBM에서 환자 진료에 초점을 둔 임상진료지침 (clinical practice guideline)의 개발은 임상의학 연구결과를 바탕으로 진행되지만, EBPH와 관련한 공중보건지침 (public health guideline, 이하 PHG) 개발에서는 더 다양한 학문영역의 연구결과들을 포함해야 하기 때문이다 [6, 15].

이상의 차이점들 때문에, EBPH 수행에 반영할 근거의 확보를 위해서는 많은 자원의

Volume: 39, Article ID: e2017030

https://doi.org/10.4178/epih.e2017030

투입과 함께 특단의 전략이 필요하다 [11]. 그 전략은 다음 세 가지로 요약된다. 첫째, 근거 생성을 위한 전담조직 운영이다. 영국의 NICE (https://www.nice.org.uk/)와 한국의 NECA (한국보건의료연구원, http://neca.re.kr/) 등이 EBPH를 추구하는 대표적인 조직이다. 둘째, 지식기반 (knowledge base) 구축이다. 공중보건을 위협하는 다양한 위험요인 (risk factors)들에 대한 정보들을 지속적으로 제공하기 위한 목적으로 운영되고 있다 [24]. The Cochrane Library (http://www.cochranelibrary.com/), Health Research Policy and Systems (https://health-policy-systems.biomedcentral.com/) 등이 대표적이며, 국내는 질병관리본부 산하 국립의과학지식센터 (http://library.nih.go.kr/ncmiklib/) 가 같은 운영 목적을 표방하고 있다. 셋째, 체계적 고찰 (systematic reviews, SR) 연구방법을 적극 활용하는 것이다 [11, 22]. EBPH에서 SR로 생성된 근거들은 공중보건사업의 효과와 영향을 측정하거나, PHG 개발에 활용된다 [25].

3. 근거중심 공중보건 활성화와 관련한 요소들

EBPH를 위해 생성된 대부분의 근거들은 서구사회에서 얻어낸 연구결과들을 기반하고 있다는 점에서 [6], 국내 공중보건 의사결정에 적용할 근거들이 상대적으로 빈약하다고 볼 수 있다. 그러나 위에서 지적한 것처럼 우리나라는 이미 NECA란 전담조직과 국립의과학지식센터란 정보기반을 갖추고 있기에, EBPH가 국내 현실에서 활성화가 되지 못하는 이유로써 근거의 상대적 부족 이외의 다른 장애 요소들을 살펴볼 필요가 있다.

Volume: 39, Article ID: e2017030

https://doi.org/10.4178/epih.e2017030

Tricco 등 [25]은 근거중심의 의사결정을 할 수 없게 만드는 장애요소들을 태도 (attitudes), 지식 (knowledge), 기술 (skills), 행위 (behavior)의 4가지 차원에서 정리하여 제시하였다. Jacobs 등 [26]은 9개의 장애요소들을 도출하였고, 이중 3가지 요소 – 근거중심 공중보건 프로그램 개발 경험 부족 (lack skills to develop evidence-based programs), 의사결정과정의 소통기술 부족 (lack skills to effectively communicate findings to state-level policy makers), 여러 영역의 전문성 한계 (feels the need to be an expert on many issues) –를 개선 가능한 (modifiable) 요소임을 제시하였다. Oliver 등 [27]은 발표된 연구결과가 시기적절하게 전달되지 못하는 점과, 전달받은 근거들을 의사결정자가 제대로 이해하지 못하는 것이 EBPH 수행의 가장 큰 장애요소임을 강조하였다. 그런데 Oliver 등 [27]의 첫째 주요 장애요소인 근거전달의 문제는 Jacobs 등 [26]의 경험부족과 소통부족 요소와 같은 의미를 가지며, 둘째 주요 장애요소인 의사결정자의 이해부족은 전문성 한계와 같은 뜻이다. 결국 근거생성의 연구자와 의사결정의 행정가 간에 원활한 소통 부재가 EBPH 활성화의 장애 요소인 것이다. Hyder 등 [28]이 EBPH 활성화를 위해 6가지 제언을 하였는데 (Table 1), 이들 모두 다 두 주체들 간의 의사소통을 원활히 하려는 방안으로 요약할 수 있다 [27].

Volume: 39, Article ID: e2017030

https://doi.org/10.4178/epih.e2017030

결론 및 제언

이상을 요약하자면, EBPH 활성화를 이루기 위해서는 우선적으로 근거생성의 연구자와 의사결정의 행정가 간에 원활한 소통이 이루어 져야 한다는 것이다. 그렇다면 2017년 질병관리본부가 추진하는 LTBI 검진사업의 대상 선정에서 있어 여러 논란과 이후 변경이 있었던 것에 있어서 [1-3], 공중보건관련 정책의 결정과 수행 주체인 질병관리본부가 주도하여 두 주체 간의 소통 수준을 평가하고, 근거에 기반한 의사결정과정에 있어 다각도의 개선책을 도출하기를 제안한다. 혹시 관련 근거에 바탕하여 개발된 PHG에 기반하지 않고 이해상충이 개입될 수 있는 관련자들 의견에 따라 의사결정이 이루어지는 구도였다면, 이를 시급히 개선해야 할 것이다. 만약 의사결정에 필요한 근거가 부족한 경우라면, 고위험군이나 소외계층을 중심으로 시범사업을 추진해서 관련 근거들을 확보한 다음, 전국민을 대상으로 보건 정책을 추진하는 것이 합당할 것이다 [6].

한편 EBPH의 연구자-행정가 간의 의사소통은, EBM의 환자-의료진 간의 의사소통보다 더 어렵다 [11]. 왜냐하면 EBPH의 의사결정 과정이 EBM과 달리 매우 독특하면서 복잡한 과정을 밟기 때문이다 [7]. 이를 극복하기 위해서 국가단위로 수립된 전략에 따라 설립된 산하 조직들의 안정적인 운영이 필요하다 [14]. 한국 정부는 정책-근거의 간극 (policy-evidence gap)을 분석하여 공중보건 의사결정에 필요한 근거들이 무엇인가를 도출하기 위하

여 [23] 한국보건의료연구원을, SR과 PHG 같이 질 높은 근거들을 창출하여 의사결정 시점에 맞게 전달하기 위하여 [6] 국립의과학지식센터를, 의사결정자들이 가치기반의 의사결정을 제대로 하도록 교육 및 훈련을 위하여 [18] 한국건강증진개발원 (www.khealth.or.kr) 을 각각 운영하고 있다. 그렇다면 국내에서 EBPH 활성화를 위하여는 이들 세 기관들을 묶는 리더십 네트워크 (leadership network)를 보다 강화하는 것이다 [29]. 이에 관하여는 STP (SUPPORT Tools for evidence-informed health Policymaking의 운영 전략을 활용하기를 제안한다 [12, 21]. 나아가 이미 국제적 공조를 갖춘 DECIDE (The Developing and Evaluating Communication Strategies to Support Informed Decisions and Practice Based on Evidence) consortium 에도 적극 참여하기를 제안한다 [30].

Volume: 39, Article ID: e2017030

https://doi.org/10.4178/epih.e2017030

References

1. Centers for Disease Control and Prevention in Korea. Tuberculosis is progressing now. [cited 2017 Jun 30]. Available from: http://cdc.go.kr/CDC/intro/CdcKrIntro0505.jsp?menuIds=HOME001-MNU1154-MNU2557-MNU2487&fid=7947&cid=73871 (korean)

2. Bae JM. An opposing view on including high school students in a latent tuberculosis infection control program in Korea. Epidemiol Health 2017;39:e2017015.

3. Centers for Disease Control and Prevention in Korea. Tuberculosis is progressing now. [cited 2017 June 30]. Available from: Decision of the third Meeting on National Committee of Tuberculosis Controls in 2017. http://www.cdc.go.kr/CDC/notice/CdcKrIntro0201.jsp?menuIds=HOME001-MNU1154-MNU0005-MNU0011&cid=75212 (korean)

4. Jenicek M. Epidemiology, evidenced-based medicine, and evidence-based public health. J Epidemiol 1997;7(4):187-97.

5. Sackett DL, Rosenberg WM, Gray JA, Haynes RB, Richardson WS. Evidence based medicine: what it is and what it isn't. BMJ 1996;312(7023):71-2.

6. Brownson RC, Fielding JE, Maylahn CM. Evidence-based public health: a fundamental concept for public health practice. Annu Rev Public Health 2009;30:175-201.

7. Jacobs JA, Jones E, Gabella BA, Spring B, Brownson RC. Tools for implementing an evidence-based approach in public health practice. Prev Chronic Dis 2012;9:E116.

8. Rychetnik L, Hawe P, Waters E, Barratt A, Frommer M. A glossary for evidence based public health. J Epidemiol Community Health 2004;58(7):538-45.

9. Baker EA, Brownson RC, Dreisinger M, McIntosh LD, Karamehic-Muratovic A. Examining

the role of training in evidence-based public health: a qualitative study. Health Promot Pract 2009;10(3):342-8.

10. Muir Gray JA. Evidence based policy making. BMJ 2004;329(7473):988-9.

11. Tang JL, Griffiths S. Review paper: epidemiology, evidence-based medicine, and public health. Asia Pac J Public Health 2009;21(3):244-51.

12. Oxman AD, Lavis JN, Lewin S, Fretheim A. SUPPORT Tools for evidence-informed health Policymaking (STP) 1: What is evidence-informed policymaking? Health Res Policy Syst 2009;7 Suppl 1:S1.

13. Heller RF, Page J. A population perspective to evidence based medicine: "evidence for population health". J Epidemiol Community Health 2002;56(1):45-7.

14. Orton L, Lloyd-Williams F, Taylor-Robinson D, O'Flaherty M, Capewell S. The use of research evidence in public health decision making processes: systematic review. PLoS One 2011;6(7):e21704.

15. Oliver K, Lorenc T, Innvær S. New directions in evidence-based policy research: a critical analysis of the literature. Health Res Policy Syst 2014;12:34.

16. Sackett DL, Straus SE, Richardson WS, Rosenberg W, Haynes RB. Evidence-based medicine: how to practice and teach EBM. 2nd ed. Edinburgh: Churchill Livingstone; 2000. p3.

17. Kohatsu ND, Robinson JG, Torner JC. Evidence-based public health: an evolving concept. Am J Prev Med 2004;27(5):417-21.

18. Brownson RC, Allen P, Duggan K, Stamatakis KA, Erwin PC. Fostering more-effective public health by identifying administrative evidence-based practices: a review of the literature. Am J Prev Med 2012;43(3):309-19.

19. Cairney P, Oliver K. Evidence-based policymaking is not like evidence-based medicine, so

Volume: 39, Article ID: e2017030

https://doi.org/10.4178/epih.e2017030

how far should you go to bridge the divide between evidence and policy? Health Res Policy Syst 2017;15(1):35.

20. Brownson RC, Gurney JG, Land GH. Evidence-based decision making in public health. J Public Health Manag Pract 1999;5(5):86-97.

21. Lavis JN, Wilson MG, Oxman AD, Grimshaw J, Lewin S, Fretheim A. SUPPORT Tools for evidence-informed health Policymaking (STP) 5: Using research evidence to frame options to address a problem. Health Res Policy Syst 2009;7 Suppl 1:S5.

22. McMichael C, Waters E, Volmink J. Evidence-based public health: what does it offer developing countries? J Public Health 2005;27(2):215-21.

23. Bowen S, Erickson T, Martens PJ, Crockett S. More than "using research": the real challenges in promoting evidence-informed decision-making. Healthc Policy 2009;4(3):87-102.

24. Thacker SB, Ikeda RM, Gieseker KE, Mendelsohn AB, Saydah SH, Curry CW, et al. The evidence base for public health informing policy at the Centers for Disease Control and Prevention. Am J Prev Med 2005;29(3):227-33.

25. Tricco AC, Cardoso R, Thomas SM, Motiwala S, Sullivan S, Kealey MR, et al. Barriers and facilitators to uptake of systematic reviews by policy makers and health care managers: a scoping review. Implement Sci 2016;11:4.

26. Jacobs JA, Dodson EA, Baker EA, Deshpande AD, Brownson RC. Barriers to evidence-based decision making in public health: a national survey of chronic disease practitioners. Public Health Rep 2010;125(5):736-42.

27. Oliver K, Innvar S, Lorenc T, Woodman J, Thomas J. A systematic review of barriers to and facilitators of the use of evidence by policymakers. BMC Health Serv Res 2014;14:2.

28. Hyder AA, Corluka A, Winch PJ, El-Shinnawy A, Ghassany H, Malekafzali H, et al. National

policy-makers speak out: are researchers giving them what they need? Health Policy Plan 2011;26(1):73-82.

29. Wright K, Rowitz L, Merkle A, Reid WM, Robinson G, Herzog B, et al. Competency development in public health leadership. Am J Public Health 2000;90(8):1202-7.

30. Treweek S, Oxman AD, Alderson P, Bossuyt PM, Brandt L, Brożek J, et al. Developing and Evaluating Communication Strategies to Support Informed Decisions and Practice Based on Evidence (DECIDE): protocol and preliminary results. Implement Sci 2013;8:6.

Volume: 39, Article ID: e2017030

https://doi.org/10.4178/epih.e2017030

Table 1. Suggested strategies to promote evidence-based public health*

Strengthening demand from policy-makers
Creating formal processes to facilitate dialogue
Improving packaging of evidence
Enhancing technical capabilities and competencies
Implementing incentives for researchers
Recognizing the role of informal relationships:

* Modified from Hyder et al. (2011) [28]
